# Supplementary material for: Tissue distribution and retention drives efficacy of rapidly clearing VHL-based PROTACs
Source: Commun Med (Lond). 2024 May 16;4:87. doi: 10.1038/s43856-024-00505-y (PMC11099041; doi:10.1038/s43856-024-00505-y)

Supplementary Information

Tissue distribution and retention drives efficacy of rapidly clearing VHL-based PROTACs

Donglu Zhang[1], Bin Ma[1], Peter S Dragovich[1], Li Ma[1], Shu Chen[1], Eugene C Chen[1], Xiaofen Ye[1], Joyce Liu[1], Jennifer Pizzano[2], Elizabeth Bortolon[2], Emily Chan[1], Xing Zhang[1], Yi-Chen Chen[1], Elizabeth S Levy[1], Robert L Yauch[1], S Cyrus Khojasteh[1], Cornelis ECA Hop[1]

[1]Genentech; 1 DNA Way, South San Francisco, CA 94080, USA

[2]Arvinas; 5 Science Park, 395 Winchester Ave, New Haven, CT 06511, USA

Supplementary Method

Synthesis of radiolabel

Supplementary Scheme 1. Radiolabel synthesis

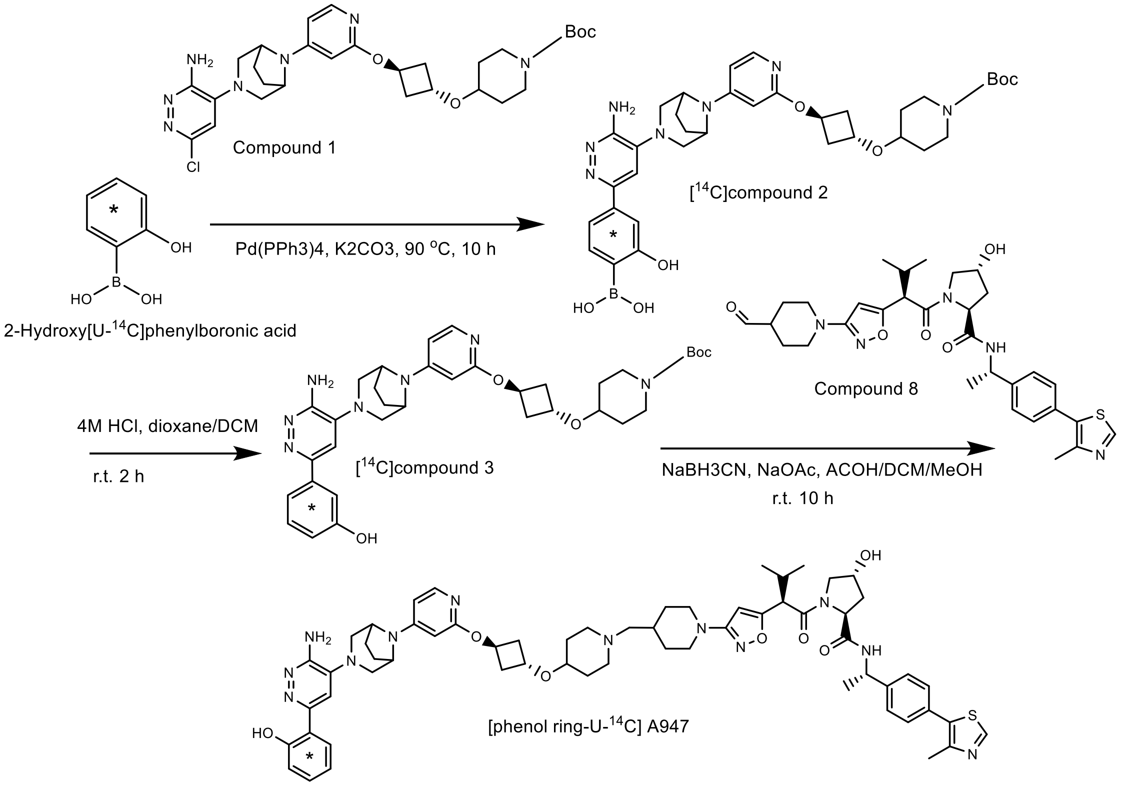

Synthesis scheme of ^{14}C-A947 was presented in Supplementary Scheme S1. To a mixture of compound **1** (713.9 mg, 1.218 mmol, 1 equivalent), 2-hydroxy[U^{14}C]phenyl boronic acid (67 mCi @ 55 mCi/mmol, 1.219 mmol), potassium carbonate (337.2 mg, 2.436 mmol, 2 eq) in dioxane (10 mL) and water (1.6 mL) degassed with argon was added in one lot, tetrakis(triphenylphosphine)-palladium(0) (142 mg, 0.122 mmol, 0.1 eq). The mixture was heated under nitrogen at 90°C overnight (16 h). The mixture was transferred to a 100 mL round

bottom flask using dioxane, evaporated to dryness, dissolved in ethyl acetate (4 mL) and ethyl acetate/hexane (2:1, 4 mL) and loaded onto a 55 g SNAP KP-NH cartridge, eluting with ethyl acetate/hexane 2:1. Fractions containing the desired material were combined and the solution was stored overnight at -20°C, warmed to room temperature and counted as 41.22 mCi, then evaporated to dryness, transferred to a 100 mL round bottom flask using dichloromethane and evaporated to give [^{14}C]compound **2** (582 mg, 41.22 mCi, 61% yield) as a light yellow solid.

To [^{14}C] compound **2** (41.2 mCi, 0.749 mmol, 1 eq) in dichloromethane (9.6 mL) was added 4 M hydrochloric acid in dioxane (9.6 mL) and the mixture was stirred at room temperature for 2 h and evaporated to dryness. Methanol (20 mL) was added to dissolve the precipitate and evaporated again to give [^{14}C]compound **3** (41.2 mCi, yield assumed quantitative) as an orange oil, referenced JJN/CFQ44370/33/1, which was used in the next step without any further purification. Thin-layer chromatography (TLC, KP-NH, ethyl acetate/methanol 9:1) showed complete conversion to [^{14}C]compound **3**.

To a solution of [^{14}C]compound **3** (41.2 mCi @55 mCi/mmol, 0.749 mmol, 1 eq) and compound **8** (444.7 mg, 0.749 mmol, 1 eq) in dichloromethane (19 mL) and methanol (19 mL) was added sodium acetate (246 mg, 2.9964 mmol, 4 eq) then acetic acid (9 drops) until pH 6. Sodium cyanoborohydride (235 mg, 3.75 mmol, 5 eq) was then added and the reaction mixture stirred at 20°C overnight. Water (25 mL) was added and the mixture was extracted with dichloromethane (3 x 25 mL). The organic extracts were dried over sodium sulfate, filtered and evaporated to give the crude material as a solid. The material was dissolved in mixtures of

dichloromethane/methanol, loaded onto a 55 g SNAP KP-NH cartridge, eluting with ethyl acetate/methanol 95:5. Fractions containing the desired product were combined to give, after evaporation and azeotrope with heptane, a light yellow solid (797.8 mg) with purity by TLC (KP-NH, ethyl acetate/methanol 9:1) of ~60-70%. The material was dissolved in dichloromethane and loaded onto a 28 g SNAP KP-NH cartridge (equilibrated with dichloromethane) and eluted with dichloromethane, dichloromethane/methanol 99:1 to 98:2. Fractions containing the desired product were combined and counted as 16.56 mCi and stored overnight at -20°C. The solution was evaporated and azeotroped with heptane, redissolved in ethyl acetate/methanol 9:1 and transferred to a 100 mL round bottom flask, which was evaporated to give a light yellow solid. The material was desiccated to constant weight over phosphorus pentoxide to give [phenol ring-U^{14}C]A947 (382.7mg, 16.56 mCi, 43.27 µCi/mg, 40% yield). The material had a radiopurity of 97.2% and chemical purity of 98.2%. The radiolabel had the same chromatography retention as the authentic non-radiolabel material. (+ESI) MS, m/z 561.2706 (Z=2) and 374.5167 (z=3). ^{1}H NMR (400 MHz, DMSO) δ 14.14 (s, 6H), 8.98 (s, 6H), 8.77 (d, *J* = 7.7 Hz, 1H), 8.39 (d, *J* = 7.7 Hz, 6H), 7.92 (q, *J* = 3.1 Hz, 7H), 7.77 (d, *J* = 6.0 Hz, 7H), 7.43 (m, *J* = 14.0 Hz, 36H), 7.22 (m, *J* = 4.2 Hz, 7H), 6.87 (d, *J* = 8.2 Hz, 11H), 6.83 (d, *J* = 1.1 Hz, 3H), 6.53 (d, *J* = 1.9 Hz, 5H), 6.52 (d, *J* = 1.8 Hz, 5H), 6.14 (d, *J* = 1.6 Hz, 7H), 6.10 (s, 6H), 5.97 (s, 12H), 5.19 (m, *J* = 3.6 Hz, 7H), 5.10 (d, *J* = 3.6 Hz, 6H), 4.99 (d, *J* = 2.8 Hz, 1H), 4.91 (m, *J* = 7.1 Hz, 8H), 4.66 (t, *J* = 7.6 Hz, 1H), 4.49 (s, 10H), 4.36 (t, *J* = 7.8 Hz, 6H), 4.28 (m, *J* = 5.8 Hz, 15H), 4.03 (q, *J* = 7.1 Hz, 13H), 3.71 (q, *J* = 5.0 Hz, 6H), 3.58 (q, *J* = 9.7 Hz, 21H), 3.42

(t, *J* = 9.2 Hz, 9H), 3.25 (d, *J* = 10.2 Hz, 18H), 3.01 (d, *J* = 11.3 Hz, 13H), 2.70 (q, *J* = 13.1 Hz, 27H), 2.24 (m, *J* = 5.4 Hz, 51H), 2.09 (d, *J* = 6.8 Hz, 12H), 1.99 (s, 25H), 1.96 (s, 13H), 1.75 (m, *J* = 6.8 Hz, 35H), 1.63 (s, 3H), 1.43 (t, *J* = 9.6 Hz, 12H), 1.38 (d, *J* = 7.0 Hz, 23H), 1.18 (t, *J* = 7.1 Hz, 20H), 1.11 (q, *J* = 9.3 Hz, 15H), 0.95 (t, *J* = 6.9 Hz, 20H), 0.83 (d, *J* = 7.6 Hz, 4H), 0.79 (d, *J* = 6.7 Hz, 17H). Mass and nuclear magnetic resonance (NMR) spectra of ^{14}C-A947 are listed in Supplementary Figure 4.

Supplementary Table 1. Individual concentrations of radioactivity in blood and plasma as determined by liquid scintillation counting at specified times after single intravenous administration of ^{14}C-A947 to male Sprague Dawley rats (4 mg/kg)

	Concentration (ng Equivalents ^{14}C-A947/g)							
	Animal (Time Point)							
Sample	R0401 (0.25 h)	R0402 (1 h)	R0403 (4 h)	R0404 (8 h)	R0405 (24 h)	R0406 (48 h)	R0407 (168 hr)	R0408 (336 h)
Blood	1130	423	242	124	79.7	49.3	9.12	5.70
Plasma	1220	453	252	140	67.9	49.9	9.54	3.56

h, Hours

Supplementary Table 2. Cumulative percent of radioactive dose in urine, feces, and bile at specified intervals after single intravenous administration of ^{14}C-A947 to male Sprague Dawley bile duct-cannulated rats (4 mg/kg)

		Percent of Radioactive Dose				
		Animal				
Sample	Time Point (h)	R0001	R0002	R0003	Mean	SD
Urine	Predose	0.00	0.00	0.00	0.00	0.00
Urine	0 - 8	1.80	1.51	1.73	1.68	0.154
Urine	0 - 24	2.10	1.96	2.02	2.03	0.0750
Urine	0 - 48	2.34	2.30	2.30	2.31	0.0253
Urine	0 - 72	2.47	2.52	2.54	2.51	0.0380
Urine	0 - 96	2.58	2.72	2.66	2.66	0.0695
Urine	0 - 120	2.65	2.88	2.82	2.78	0.120
Urine	0 - 144	2.76	3.08	3.00	2.95	0.163
Urine	0 - 168	2.88	3.27	3.17	3.10	0.202
Feces	Predose	0.00	0.00	0.00	0.00	0.00
Feces	0 - 8	0.0368	0.0313	0.490	0.186	0.263
Feces	0 - 24	3.09	3.48	2.79	3.12	0.346
Feces	0 - 48	3.68	4.27	3.51	3.82	0.400
Feces	0 - 72	4.10	4.67	3.91	4.23	0.399
Feces	0 - 96	4.36	4.95	4.27	4.53	0.371
Feces	0 - 120	4.60	5.21	4.58	4.80	0.360
Feces	0 - 144	4.88	5.47	4.85	5.07	0.353
Feces	0 - 168	5.09	5.75	5.05	5.30	0.396
Bile	Predose	0.00	0.00	0.00	0.00	0.00
Bile	0 - 8	13.8	14.7	14.1	14.2	0.463
Bile	0 - 24	28.3	27.5	26.7	27.5	0.817
Bile	0 - 48	38.3	37.9	37.2	37.8	0.563
Bile	0 - 72	44.2	43.8	43.2	43.8	0.495
Bile	0 - 96	48.2	47.3	47.0	47.5	0.612
Bile	0 - 120	51.1	49.7	49.8	50.2	0.823
Bile	0 - 144	53.7	51.8	51.8	52.4	1.06
Bile	0 - 168	55.8	53.2	53.5	54.2	1.42
Carcass	168	28.0	32.3	35.6	32.0	3.79
	Subtotal	28.0	32.3	35.6	32.0	3.79
Total		92.3	94.9	97.6	95.0	2.66

h, Hours
SD, standard deviation.

Supplementary Table 3. Cumulative percent of radioactive dose in urine, and feces at specified intervals after single intravenous administration of ^{14}C-A947 to male Sprague Dawley rats (4 mg/kg)

Sample	Time Point (h)	Percent of Radioactive Dose				
		Animal			Mean	SD
		R0204	R0205	R0407		
Urine	Predose	0.00	0.00	0.00	0	0
Urine	0 - 24	1.83	1.62	1.38	1.61	0.23
Urine	0 - 48	2.02	1.84	1.54	1.80	0.24
Urine	0 - 72	2.14	1.96	1.67	1.92	0.24
Urine	0 - 96	2.23	2.03	1.77	2.01	0.23
Urine	0 - 120	2.32	2.08	1.84	2.08	0.24
Urine	0 - 144	2.38	2.13	1.90	2.14	0.24
Urine	0 - 168	2.44	2.18	1.95	2.19	0.25
Feces	Predose	0.00	0.00	0.00	0.00	0.00
Feces	0 - 24	36.5	41.2	48.0	41.90	5.78
Feces	0 - 48	54.8	59.2	64.0	59.33	4.60
Feces	0 - 72	62.4	66.5	72.7	67.20	5.19
Feces	0 - 96	66.9	69.4	77.0	71.10	5.26
Feces	0 - 120	70.1	71.9	80.1	74.03	5.33
Feces	0 - 144	72.0	74.5	82.0	76.17	5.20
Feces	0 - 168	74.0	76.4	83.5	77.97	4.94
Total		76.44	75.58	85.45	79.16	5.47

h, Hours
SD, standard deviation.

Supplementary Table 4. Concentrations of radioactivity in plasma and excised tissues at specific times after intravenous administration of ^{14}C-A947 to rats (4 mg/kg, 200 µCi/kg)

Sample	Concentration (ng Equivalents ^{14}C-A947 /g)				
	Animal				
	R0201 (2 h)	R0202 (24 h)	R0203 (48 h)	R0204 (168 h)	R0205 (336 h)
Plasma (sac)	385	74.6	36.9	10.7	3.72
Adrenal gland (Left)	23800	15000	10200	5610	2770
Adrenal gland (Right)	24300	12900	11900	5340	2990
Bladder (urinary)	896	720	414	680	89.6
Bone marrow-Femur	6760	5580	4380	2960	1620
Bone-Femur	2100	1470	822	569	376
Brain	64.8	63.9	55.8	BLQ	BLQ
Contents Large intestine	615	4210	1660	290	153
Contents Small intestine	6300	879	186	64.6	13.8
Contents Stomach	60.9	6.03	1.76	20.5	1.93
Esophageal contents	2.95	BLQ	BLQ	BLQ	BLQ
Esophagus	2150	1290	580	243	103
Eye (Left)	1040	705	355	227	125
Eye (Right)	1090	543	374	203	107
Fat (reproductive)	616	390	379	290	114
Heart	7000	2520	1690	687	270
Kidney (Left)	24100	15900	11600	5860	2110
Kidney (Right)	25400	16900	11700	5860	2080
Large intestine	2150	1160	671	202	BLQ
Liver	30200	13300	9230	5610	1500
Lungs	16800	5260	3210	1650	607
Lymph node(s)	4680	3680	3430	5420	1110
Muscle (thigh)	1200	832	502	136	BLQ
Pancreas	9460	6540	5590	2170	1210
Prostate gland	1440	1180	1140	1610	594
Salivary gland(s)	8810	7030	5010	3900	1180
Skin (dorsal shaved)	367	208	158	72.4	45.9
Small intestine	3780	1330	58.1	215	211
Spleen	20700	13100	12400	12000	4120
Stomach	1680	880	625	201	132
Testis (Left)	105	94.1	68.2	61.4	52.5
Testis (Right)	87.1	76.5	73.3	83.3	58.1
Thymus	1260	1340	1110	1620	788
Thyroid	13000	11900	13900	12100	6820

BLQ Below the limit of quantitation.
h Hours.

Supplementary Table 5. Excised Tissue:plasma concentration ratios at specific times after intravenous administration of ^{14}C-A947 to rats (4 mg/kg, 200 µCi/kg)

Sample	Excised Tissue:Plasma Concentration Ratio				
	Animal				
	R0201 (2 h)	R0202 (24 h)	R0203 (48 h)	R0204 (168 h)	R0205 (336 h)
Adrenal gland (Left)	61.9	202	275	525	745
Adrenal gland (Right)	63.2	173	323	500	804
Bladder (urinary)	2.33	9.65	11.2	63.6	24.1
Bone marrow-Femur	17.6	74.9	119	277	435
Bone-Femur	5.44	19.7	22.3	53.2	101
Brain	0.168	0.857	1.51	N.A.	N.A.
Contents Large intestine	1.60	56.5	45.1	27.1	41.2
Contents Small intestine	16.3	11.8	5.03	6.04	3.70
Contents Stomach	0.158	0.081	0.048	1.92	0.518
Esophageal contents	0.008	N.A.	N.A.	N.A.	N.A.
Esophagus	5.57	17.3	15.7	22.8	27.7
Eye (Left)	2.71	9.45	9.62	21.3	33.5
Eye (Right)	2.82	7.29	10.1	19.0	28.8
Fat (reproductive)	1.60	5.24	10.3	27.1	30.5
Heart	18.2	33.8	45.9	64.3	72.4
Kidney (Left)	62.5	214	314	549	567
Kidney (Right)	65.9	227	317	549	560
Large intestine	5.58	15.6	18.2	18.9	N.A.
Liver	78.4	178	250	525	404
Lungs	43.7	70.6	87.0	154	163
Lymph node(s)	12.1	49.4	93.0	508	297
Muscle (thigh)	3.13	11.2	13.6	12.7	N.A.
Pancreas	24.6	87.8	151	203	324
Prostate gland	3.73	15.8	31.0	151	160
Salivary gland(s)	22.9	94.3	136	365	316
Skin (dorsal shaved)	0.954	2.79	4.29	6.78	12.3
Small intestine	9.81	17.8	1.57	20.1	56.7
Spleen	53.6	176	337	1120	1110
Stomach	4.35	11.8	16.9	18.8	35.5
Testis (Left)	0.273	1.26	1.85	5.75	14.1
Testis (Right)	0.226	1.03	1.99	7.80	15.6
Thymus	3.26	18.0	30.0	152	212
Thyroid	33.9	160	376	1130	1830

h Hours.
N.A. Not applicable.

Supplementary Table 6. Metabolite identification in urine, bile, and feces of rats after intravenous administration of ^{14}C-A947 (4 mg/kg, 200 µCi/kg)

Metabolite	Accurate Mass	Retention min	Major Fragments	Reaction	Matrix	%Rad in bile	%rad in urine	%rad in feces
Parent	1121.5770 561.2925	28.25	920.5137, 578.2794, 544.3033, 460.2918, 391.1875, 377.2182, 247.1440, 202.0685		B, F, U	23.11	20.6	18.95
M1	1201.5257 601.2674*	14.65	299.1391	Sulfation	B, U, F	7.34	0.2	3.9
M2	493.2206	14.65	391.1878	di-Oxidation (+2O)	B, U, F	7.34	1.46	7.17
M3	610.2697	15.15	364.1885,346.1765, 279.1335, 202.0686	N-dealkylation+ oxidation (+O)	B, U			
M4	1201.5257 601.2674*	15.8		Sulfation	B, U, F	0.2		0.2
M5	391.1882	16.78	189.1026, 167.0339, 149.0234, 71.0863	O-dealkylation	B, U, F	1.49	7.53	1.86
M6	849.3716	16.70	Not triggered	M10+GSH	B, F			
M7	1297.6091 649.3085	19.35	578.2795, 544.3046 391.1871,377.2184, 202.0687	Glucuronidation		6.78	2.93	
M8	921.5004 461.2540	19.85	391.1868, 378.2018 376.1687, 344.1964, 84.0814	Hydrolysis	B, F	3.47		
M9	808.4522 404.7297	20.62		Hydrolysis	B, F	3.08		
M10	544.3036	20.88	490.3000, 391.1874, 162.1125, 84.0814	N-dealkylation	B, F	4.48	3.66	
M11	461.2304	21.77	391.1879,189.1030,157.1014	O-dealkylation	B, F	2.79	2.93	4.34
M12	1137.5720	23.95	Not triggered	Oxidation (+O)	B, U, F	1.88		1.1
M13	1123.5928 562.3000	25.68	792.4566, 708.3981, 641.3932, 544.3025, 391.1879, 332.2333, 249.1598, 248.1759, 247.1441, 202.0688, 98.0969, 96.0813	Reduction (+2H)	B, U, F	low	low	25.33
M13a	1123.5928 562.3000	26.12	from 562: 354.7026, 355.0241, 391.1871, 248.1754 , 202.0681	Reduction (+2H)				7.53
M14	1137.5720	25.41	1041.4065, 594.2730, 592.2589, 391.1889	Oxidation (+O)	B, U, F	5.89		
M15	1137.5720	26.38	594.2763, 544.3025, 391.1878, 377.2184, 375.2037, 218.0636	Oxidation (+O)	B, F	4.51		3
M16	1137.5720	28.25	Not triggered	Oxidation (+O)	B			
M17	1473.642 737.3251	16.44	920.5057, 578.2795, 544.3022, 460.2984, 391.1863, 377.2164, 375.2016, 202.0686	Diglucuronidatio n	B, U		21.86	
M18	572.2989	24.41	278.6552*(556.3031), 391.1879, 247.1437	Dealkylation	B	6.31		

*****Z=2 double charged ion

Supplementary Table 7. System suitability and nonspecific binding of ^{14}C-Warfarin (3000 ng/mL) in control rat plasma or DPBS after dialysis at 37°C for 7 hours

Radioactivity Concentration of ^{14}C-Warfarin							
Receiver dpm/mL		Donor dpm/mL		Percent	Percent	Fu	Percent
Individual	Mean	Individual	Mean	Bound	Unbound		Recovered
Plasma (Suitability)							
4130	3600	243000	247000	98.5	1.46	0.015	91.9
3620		248000					
3020		250000					
DPBS (NSB)							
266000	266000	245000	245000	NA	NA	NA	84.8
NA[a]		NA					
NA[a]		NA					

DPBS Dulbecco's phosphate-buffered saline.
Fu Fraction unbound: Percent unbound/100.
NA Not applicable.
NSB Nonspecific binding.
a Insufficient volume, only 1 replicate run.

Supplementary Table 8. System suitability and nonspecific binding of ^{14}C-A947 (25,000 dpm/mL) in control rat plasma or DPBS after dialysis at 37°C for 7 hours

Radioactivity Concentration of ^{14}C-A947							
Receiver dpm/mL		Donor dpm/mL		Percent Bound	Percent Unbound	Fu	Percent Recovered
Individual	Mean	Individual	Mean				
Plasma (Suitability)							
190	187	25600	25400	99.3	0.734	0.007	87.4
180		25200					
190		25500					
DPBS (NSB)							
2530	2671	4100	3710	NA	NA	NA	22.8
2780		3600		NA			
2680		3430		NA			

DPBS Dulbecco's phosphate-buffered saline.
Fu Fraction unbound: Percent unbound/100.
NA Not applicable.
NSB Nonspecific binding.

Supplementary Table 9. Percentages of bound and unbound ^{14}C-A947 in excised rat tissues after intravenous administration of ^{14}C-A947 (4 mg/kg, 200 µCi/kg) and plasma following dialysis at 37°C for 7 hours

			Percentage of ^{14}C-A947				
	Initial dpm/mL		Unbound			Bound	
Tissue	Individual	Mean	Individual	Mean	SD[a]	Individual	Mean
Kidney	265280	263000	0.0402	0.0425	0.00234	100	100
	261220		0.0449			100	
	262760		0.0423			100	
Liver	226840	222000	0.0366	0.0298	0.00595	100	100
	220400		0.0266			100	
	218260		0.0261			100	
Lungs	80800	89300	0.085	0.090	0.077	99.5	99.5
	92600		0.080			99.5	
	94400		0.105			99.4	
Pancreas	103500	10100	0.00992	0.0125	0.00230	100	100
	98920		0.0132			100	
	100000		0.0143			100	
Prostate Gland	8740	8760	0.175	0.228	0.0871	99.8	99.8
	8920		0.329			99.7	
	8620		0.181			99.8	
Spleen	51900	50800	0.035	0.037	0.097	99.2	99.1
	50100		0.036			99.2	
	50300		0.042			99.0	
Thymus	10200	10100	0.0444	0.0426	0.0196	100	100
	9860		0.0612			99.9	
	10120		0.0221			100	
Plasma (4 hour)	4100	3830	0.610	0.548	0.320	96.5	96.8
	3200		0.534			96.9	
	4200		0.501			97.1	
Plasma (24 hour)	2800	2400	0.554	0.595	NA	98.4	98.2
	2300		0.637			98.1	
	2100		NA[b]			NA[b]	
Plasma (48 hour)	1800	1630	1.14	0.863	0.991	96.7	97.5
	1600		0.481			98.6	
	1500		0.971			97.1	
Control Plasma	26600	27000	0.661	0.795	0.134	99.3	99.2
	27600		0.795			99.2	
	31060		5.43			94.6	

SD Standard deviation.
NA Not applicable.
a Standard deviation applies to both bound and unbound percentages.
b Insufficient sample volume for replicate.

Supplementary Table 10. Individual tumor volume data of HCC2302 model in CB.17-SCID female mice treated with A947 (WF-31)

Day	Vehicle										Mean	SD
0.0	136.2	155.0	155.7	110.3	138.0	138.3	155.5	136.0	111.0	158.2	139.42	17.64
3.0	184.7	212.9	198.9	130.6	170.0	183.7	213.2	186.9	160.9	220.3	186.21	27.42
7.0	302.8	304.4	317.2	183.5	255.5	259.7	272.7	230.7	205.3	314.9	264.67	46.85
10.0	375.2	363.9	397.3	263.2	266.7	352.2	317.5	274.4	278.5	386.8	327.57	53.49
13.0	425.5	431.8	445.5	313.4	304.7	399.8	359.2	323.6	327.0	494.9	382.54	65.91
14.0	457.9	497.2	512.3	357.6	391.4	448.9	464.2	374.8	358.8	539.9	440.30	66.21
21.0	474.3	542.8	538.0	442.2	442.2	531.0	526.5	430.0	402.8	634.8	496.46	70.45
Day	A947 (20 mg/kg, IV, QW)										Mean	SD
0.0	131.0	144.0	151.4	162.9	118.7	150.4	130.4	164.9	118.9	144.3	141.69	16.53
3.0	185.9	191.2	236.7	232.0	186.5	175.8	170.5	235.8	172.2	188.3	197.49	26.70
7.0	209.8	241.9	322.9	334.3	219.1	220.5	241.0	346.3	199.9	199.9	253.56	57.90
10.0	223.9	270.3	361.6	390.3	244.8	273.7	238.9	390.5	203.9	188.5	278.64	75.56
13.0	277.0	331.2	433.3	436.3	292.8	297.0	275.7	532.5	238.5	211.6	332.59	101.94
14.0	297.7	329.8	491.2	493.4	301.1	321.5	304.3	580.7	252.4	204.7	357.68	121.20
21.0	336.8	348.3	510.0	552.0	323.8	348.1	336.8	655.5	270.7	239.4	392.14	133.97
Day	A947 (40 mg/kg, IV, QW)										Mean	SD
0.0	129.9	149.1	144.4	119.0	166.2	149.0	127.3	119.8	146.6	167.3	141.86	17.44
3.0	177.3	174.8	213.6	169.8	197.5	164.9	171.0	162.9	167.7	194.6	179.41	16.79
7.0	179.0	199.8	206.2	153.3	237.8	N/A	180.7	191.7	256.0	221.4	202.88	31.72
10.0	128.4	202.9	184.1	155.5	240.6	N/A	167.9	182.4	249.5	223.1	192.71	40.10
13.0	137.9	213.8	221.9	155.9	265.8	N/A	190.3	157.5	235.4	256.4	203.88	46.10
14.0	140.8	243.8	212.6	173.5	283.6	N/A	184.0	150.4	198.5	305.2	210.27	57.13
21.0	138.1	259.2	177.2	175.4	291.3	N/A	216.3	147.6	153.8	281.2	204.46	59.54

N/A, not available; IV, intravenous; QW, once a week; SD, standard deviation

Supplementary Table 11. Individual body weight data of HCC2302 model in CB.17-SCID female mice treated with A947 (WF-31)

Day	Vehicle										Mean	SD
0.0	19.0	19.0	19.0	16.0	18.0	18.0	19.0	18.0	17.0	19.0	18.20	1.03
3.0	19.0	19.0	19.0	15.0	17.0	17.0	19.0	19.0	18.0	18.0	18.00	1.33
7.0	20.0	19.0	19.0	15.0	18.0	18.0	20.0	19.0	18.0	19.0	18.50	1.43
10.0	20.0	19.0	19.0	15.0	18.0	17.0	21.0	20.0	18.0	19.0	18.60	1.71
13.0	20.0	19.0	19.0	15.0	18.0	17.0	21.0	19.0	18.0	19.0	18.50	1.65
14.0	20.0	20.0	19.0	15.0	18.0	18.0	21.0	20.0	18.0	19.0	18.80	1.69
21.0	21.0	20.0	20.0	16.0	19.0	18.0	21.0	21.0	19.0	19.0	19.40	1.58
Day	A947 (20 mg/kg, IV, QW)										Mean	SD
0.0	19.0	18.0	19.0	18.0	20.0	20.0	18.0	19.0	20.0	19.0	19.00	0.82
3.0	19.0	18.0	19.0	18.0	21.0	19.0	18.0	19.0	20.0	19.0	19.00	0.94
7.0	19.0	19.0	20.0	18.0	21.0	19.0	18.0	20.0	20.0	19.0	19.30	0.95
10.0	19.0	20.0	19.0	18.0	21.0	20.0	17.0	20.0	20.0	19.0	19.30	1.16
13.0	20.0	19.0	19.0	18.0	21.0	20.0	17.0	20.0	19.0	19.0	19.20	1.14
14.0	20.0	19.0	20.0	18.0	21.0	20.0	17.0	20.0	19.0	19.0	19.30	1.16
21.0	19.0	19.0	20.0	19.0	21.0	20.0	18.0	20.0	20.0	19.0	19.50	0.85
Day	A947 (40 mg/kg, IV, QW)										Mean	SD
0.0	20.0	19.0	19.0	20.0	20.0	19.0	19.0	19.0	20.0	19.0	19.40	0.52
3.0	20.0	17.0	18.0	19.0	18.0	19.0	19.0	19.0	19.0	18.0	18.60	0.84
7.0	20.0	17.0	18.0	20.0	18.0	N/A	19.0	19.0	20.0	17.0	18.67	1.22
10.0	20.0	17.0	18.0	20.0	18.0	N/A	19.0	19.0	19.0	18.0	18.67	1.00
13.0	20.0	18.0	18.0	20.0	19.0	N/A	19.0	19.0	18.0	18.0	18.78	0.83
14.0	20.0	18.0	17.0	20.0	19.0	N/A	19.0	19.0	18.0	17.0	18.56	1.13
21.0	20.0	18.0	15.0	19.0	19.0	N/A	19.0	20.0	15.0	14.0	17.67	2.35

N/A, not available; IV, intravenous; QW, once a week; SD, standard deviation

Supplementary Table 12. Individual tumor volume data of HCC2302 model in CB.17-SCID female mice treated with A947 (WF-34)

Day	Vehicle										Mean	SD
0.0	135.0	121.5	148.0	134.6	146.9	146.1	138.7	148.2	121.9	134.0	137.48	10.09
4.0	181.0	156.2	185.0	171.6	180.1	206.1	177.8	234.5	146.0	159.4	179.76	25.59
8.0	259.6	198.1	236.2	245.3	235.1	242.1	208.3	282.4	175.4	193.7	227.61	32.99
11.0	311.5	236.6	318.8	307.0	328.9	315.3	256.9	343.3	227.1	248.7	289.41	42.40
15.0	414.6	274.6	358.6	349.9	370.3	349.2	302.3	394.9	274.2	282.6	337.11	50.85
18.0	472.6	333.9	445.0	377.4	435.1	406.5	356.1	467.6	312.5	320.5	392.71	60.91
22.0	535.2	382.4	511.3	396.2	497.3	461.9	414.4	477.8	359.8	344.3	438.05	67.31
25.0	582.3	430.0	601.3	431.3	543.4	513.1	494.4	524.2	394.0	362.6	487.65	80.22
29.0	646.2	494.2	671.9	488.3	644.8	612.1	616.4	676.9	467.7	416.5	573.50	96.31
Day	A947 (40 mg/kg, IV, Q2W)										Mean	SD
0.0	139.0	148.8	146.1	134.0	124.0	124.9	149.0	139.4	131.5	145.1	138.18	9.31
4.0	159.7	211.4	186.7	164.2	135.2	139.3	179.9	201.7	153.3	174.4	170.59	25.14
8.0	167.2	187.8	226.7	183.9	154.9	162.0	200.6	207.8	169.1	191.5	185.15	22.50
11.0	213.5	206.1	312.2	195.5	203.5	178.8	248.2	269.4	192.7	222.2	224.20	40.98
15.0	174.8	197.2	330.0	180.2	222.6	194.4	274.2	159.2	167.0	287.6	218.71	58.61
18.0	171.3	207.7	373.1	190.3	291.8	196.5	273.5	194.8	171.1	266.1	233.60	65.58
22.0	215.0	N/A	438.6	217.6	311.3	218.3	292.1	206.8	176.3	307.8	264.87	81.42
25.0	195.9	N/A	486.6	246.5	368.2	229.9	336.4	231.1	225.7	335.7	295.10	94.03
29.0	229.9	N/A	575.6	262.5	452.3	234.1	340.9	253.2	260.5	383.8	332.50	118.72
Day	A947 (40 mg/kg, IV, QW)										Mean	SD
0.0	125.0	129.6	143.7	139.5	157.5	142.9	129.5	140.5	125.9	159.2	139.32	12.19
4.0	153.0	147.7	197.7	143.5	172.1	165.5	212.3	164.2	127.0	206.0	168.88	28.36
8.0	189.8	157.0	208.0	190.7	196.7	N/A	201.7	246.9	148.9	227.0	196.28	30.68
11.0	205.7	180.3	202.4	168.2	182.9	N/A	199.4	280.2	165.1	249.2	203.69	38.20
15.0	267.7	208.9	224.1	256.7	157.4	N/A	246.3	294.9	184.9	317.9	239.87	51.54
18.0	240.4	204.7	206.4	279.1	167.8	N/A	225.4	325.5	208.4	306.9	240.51	52.58
22.0	299.3	N/A	239.9	278.4	172.3	N/A	270.6	312.8	259.5	363.1	274.47	55.85
25.0	297.4	N/A	277.6	286.0	178.0	N/A	268.7	352.7	276.2	374.0	288.81	59.01
29.0	326.0	N/A	313.1	355.0	221.8	N/A	293.4	358.4	331.4	435.7	329.33	60.90

N/A, not available; IV, intravenous; QW, once a week; SD, standard deviation

Supplementary Table 13. Individual body weight data of HCC2302 model in CB.17-SCID female mice treated with A947 (WF-34)

Day	Vehicle										Mean	SD
0.0	20.0	21.0	20.0	22.0	20.0	23.0	21.0	21.0	22.0	19.0	20.90	1.20
4.0	21.0	21.0	21.0	21.0	21.0	23.0	21.0	21.0	22.0	19.0	21.10	0.99
8.0	20.0	22.0	21.0	21.0	21.0	23.0	22.0	21.0	22.0	19.0	21.20	1.14
11.0	21.0	22.0	21.0	21.0	22.0	23.0	22.0	21.0	23.0	20.0	21.60	0.97
15.0	21.0	22.0	21.0	22.0	23.0	24.0	22.0	21.0	23.0	20.0	21.90	1.20
18.0	21.0	21.0	20.0	20.0	21.0	22.0	22.0	20.0	23.0	19.0	20.90	1.20
22.0	22.0	22.0	20.0	22.0	22.0	23.0	22.0	21.0	23.0	21.0	21.80	0.92
25.0	22.0	21.0	21.0	22.0	23.0	23.0	22.0	22.0	23.0	21.0	22.00	0.82
29.0	22.0	21.0	21.0	22.0	23.0	24.0	23.0	22.0	23.0	21.0	22.20	1.03
Day	A947 (40 mg/kg, IV, Q2W)										Mean	SD
0.0	22.0	22.0	20.0	21.0	22.0	19.0	22.0	23.0	20.0	19.0	21.00	1.41
4.0	21.0	21.0	19.0	19.0	21.0	17.0	20.0	22.0	20.0	19.0	19.90	1.45
8.0	22.0	22.0	20.0	20.0	22.0	18.0	21.0	23.0	21.0	20.0	20.90	1.45
11.0	23.0	23.0	21.0	22.0	23.0	19.0	22.0	22.0	22.0	21.0	21.80	1.23
15.0	23.0	21.0	21.0	23.0	24.0	20.0	22.0	23.0	22.0	21.0	22.00	1.25
18.0	22.0	18.0	20.0	20.0	21.0	18.0	22.0	22.0	20.0	20.0	20.30	1.49
22.0	24.0	N/A	21.0	22.0	22.0	19.0	22.0	23.0	22.0	20.0	21.67	1.50
25.0	25.0	N/A	21.0	22.0	23.0	20.0	23.0	23.0	22.0	21.0	22.22	1.48
29.0	25.0	N/A	22.0	23.0	24.0	19.0	23.0	24.0	21.0	21.0	22.44	1.88
Day	A947 (40 mg/kg, IV, QW)										Mean	SD
0.0	21.0	23.0	21.0	21.0	21.0	20.0	22.0	21.0	21.0	20.0	21.10	0.88
4.0	20.0	22.0	21.0	20.0	20.0	18.0	21.0	19.0	21.0	20.0	20.20	1.14
8.0	20.0	21.0	22.0	21.0	21.0	N/A	22.0	21.0	22.0	21.0	21.22	0.67
11.0	20.0	22.0	22.0	21.0	21.0	N/A	22.0	21.0	22.0	21.0	21.33	0.71
15.0	20.0	22.0	22.0	22.0	21.0	N/A	23.0	22.0	23.0	22.0	21.89	0.93
18.0	19.0	20.0	21.0	19.0	20.0	N/A	21.0	21.0	21.0	21.0	20.33	0.87
22.0	20.0	N/A	22.0	20.0	21.0	N/A	23.0	21.0	22.0	21.0	21.25	1.04
25.0	20.0	N/A	22.0	21.0	21.0	N/A	24.0	21.0	22.0	21.0	21.50	1.20
29.0	20.0	N/A	23.0	21.0	21.0	N/A	22.0	20.0	21.0	22.0	21.25	1.04

N/A, not available; IV, intravenous; QW, once a week; SD, standard deviation

Supplementary Table 14. Hepatocyte uptake data of A947 in the presence and absence of rifamycin SA

Time (second)	37°C, 1 μM A947			37°C, 1 μM A947 + 1mM Rifamycin			4°C, 1 μM A947		
25	0.1101	0.14269	0.11774	0.14413393	0.18377	0.1570159			
31	0.33429	0.30989	0.26563	0.1548979	0.14942	0.3262967			
35							0.1011	0.03556	0.10956
70	0.24287	0.24316	0.25699	0.22092712	0.25976	0.2447965			
90	0.59435	0.64771	0.66881	0.17121599	0.17026	0.1886432			
110							0.06689	0.0696	0.21705
164							0.05693	0.07168	0.12192
183		1.26364	1.39211	0.38297873	0.43092	0.3908587			
306	1.06085	1.00351	1.14722	0.34355193	0.3478	0.3094001			
328							0.17888	0.18701	0.21135
Time (second)	37°C, 1 μM A947			37°C, 1 μM A947 + 1 mM Rifamycin			4°C, 1 μM A947		
	Mean	SD	N	Mean	SD	N	Mean	SD	N
25	0.12351	0.01704	3	0.16163997	0.02022	3			
31	0.30327	0.03481	3	0.21020328	0.10058	3			
35							0.08207	0.0405	3
70	0.24767	0.00807	3	0.24182883	0.01959	3			
90	0.63696	0.03838	3	0.17670672	0.01035	3			
110							0.11785	0.08593	3
164							0.08351	0.03407	3
183	1.32787	0.09085	2	0.40158483	0.02571	3			
306	1.07052	0.07234	3	0.33358385	0.02105	3			
328							0.19241	0.0169	3

Supplementary Table 15. Estimated relative distribution percent of mass spectral abundance following incubations of A947 with human and rat hepatocytes and liver microsomes

Metabolite	Accurate Mass *z=2	Retention Time (min)	Reaction	Hepatocytes		Liver Microsomes	
				Human	Rat	Human (-NADPH, -UDPGA)	Human (+NADPH, +UDPGA)
Parent	561.2911*	24.2		93.8	94.4	99.1	96.9
M2	493.2194	12.5	*O*-dealkylation + oxidation (+2O)	0.1	0.6		
M3	610.2697	13.5	*N*-dealkylation + oxidation (+O)	0.04	0.2		
M5	391.1878	14.00	*O*-dealkylation	0.1			
M7	649.3072*	17.8	Glucuronidation	0.1			
M10	544.3032	16.9	*N*-dealkylation	2.6[a]	2.2	0.3	0.5
M11	461.2297	17.9	*O*-dealkylation	0.8[a]	0.8	0.4	0.6
M15	569.2887*	22.8	Oxidation (+O)		0.02		
M16	569.2883*	24.2	Oxidation (+O)	1.1[a]	0.4		1.3
M22	649.3073*	18.5	Glucuronidation	0.4			
M23	569.2887*	22.00	Oxidation (+O)	0.2[a]	1	0.07	0.2
M24	569.2888*	22.6	Oxidation (+O)	0.1[a]	0.1	0.1	0.1
M26	570.2964*	23.2	Hydrolysis		0.3		
M27	560.2835*	26.2	Oxidation (-2H)	0.7			0.3
M29	569.2889*	21.7	Oxidation (+O)				0.07

[a]M10, M11, M16, M23, and M24 were also detectable in the hepatocyte buffer control. Rat liver microsomes produced similar metabolites to human liver microsomes.

Supplementary Table 16. Direct inhibition of P450 enzymes by A947 in HLM incubations

Compound	P450 and Substrates	IC50 (µM)
A947	CYP1A2 (Tacrine)	>10
	CYP2C8 (Paclitaxel)	>10
	CYP2C9 (Warfarin)	>10
	CYP2C19 (Mephenytoin)	3.0
	CYP2D6 (Dextromethorphan)	>10
	CYP3A4/5 (Testosterone)	>10
	CYP3A4/5 (Midazolam)	0.54

Supplementary Table 17. Time-dependent inhibition of P450 enzymes by A947 in HLM incubations

Compound	P450 and Substrates	%AUC Shift
A947	CYP1A2 (Phenacetin)	-6.7
	CYP3A4/5 (Testosterone)	38
	CYP3A4/5 (Midazolam)	33

Supplementary Table 18. Physicochemical properties of selected PROTACs

E3 Ligase	Compound	MW	cLogP	HBD	HBA	PSA	nRotB
MDM2	Average (n=2)*	1192 (1174-1210)	10.1 (9.1-11)	4.0 (4)	19 (16-22)	234 (200-268)	29 (27-31)
IAP	Average (n=4)*	1085 (1044-1122)	8.0 (5.7-11.4)	3.5 (3-4)	17 (15-20)	201 (182-228)	30 (27-33)
VHL	Average (n=13)*	1034 (949-1159)	6.6 (4.2-8.7)	4.2 (3-5)	17 (14-19)	211 (177-233)	26 (21-35)
	A-947	1121	8.4	5	19	224	17
	A-005	1119	3.7	7	20	267	10
CRBN	Average (n=19)*	903 (614-1426)	4.1 (0.4-7.7)	3.4 (1-6)	18 (11-27)	220 (117-347)	20 (10-29)
	ARV-110	812	5.2	2	15	181	9
	ARV-471	724	7.0	2	9	96	7
	CFT8634	711	3.2	2	11	108	9

*The data is from Edmondson SD, et al [8]. The average values of each physicochemical property are listed with the range in the bracket.

Supplementary Figure 1. Tissue distribution of ^{14}C-A947 in rats following intravenous administration (4 mg/kg and 200 µCi/kg) by quantitative whole-body autoradiograph at a) 24 hours, b) 168 hours. The blood calibration standards were approximately 2300, 750, 250, 75, 25, 5, and 1.5 nCi/g.

a. 24h

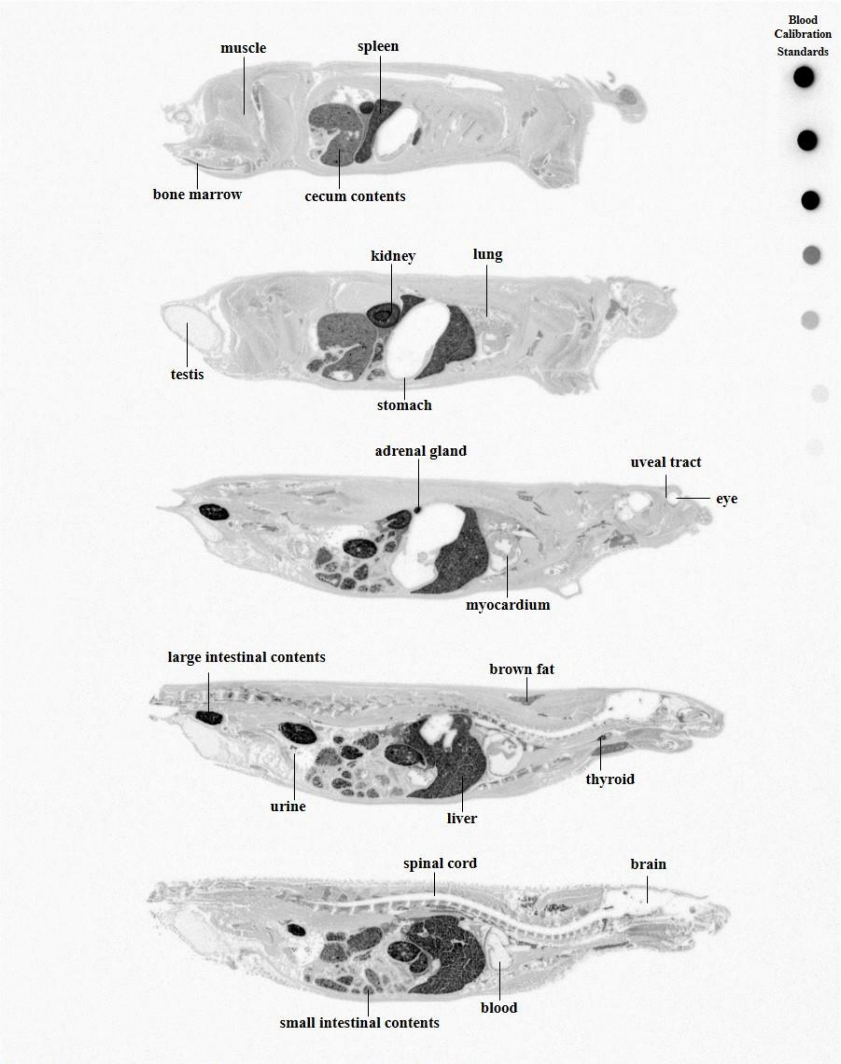

Note: Cecum and large intestinal contents are identified separately in this image; however, concentrations were averaged for quantitation.

b. 168 h

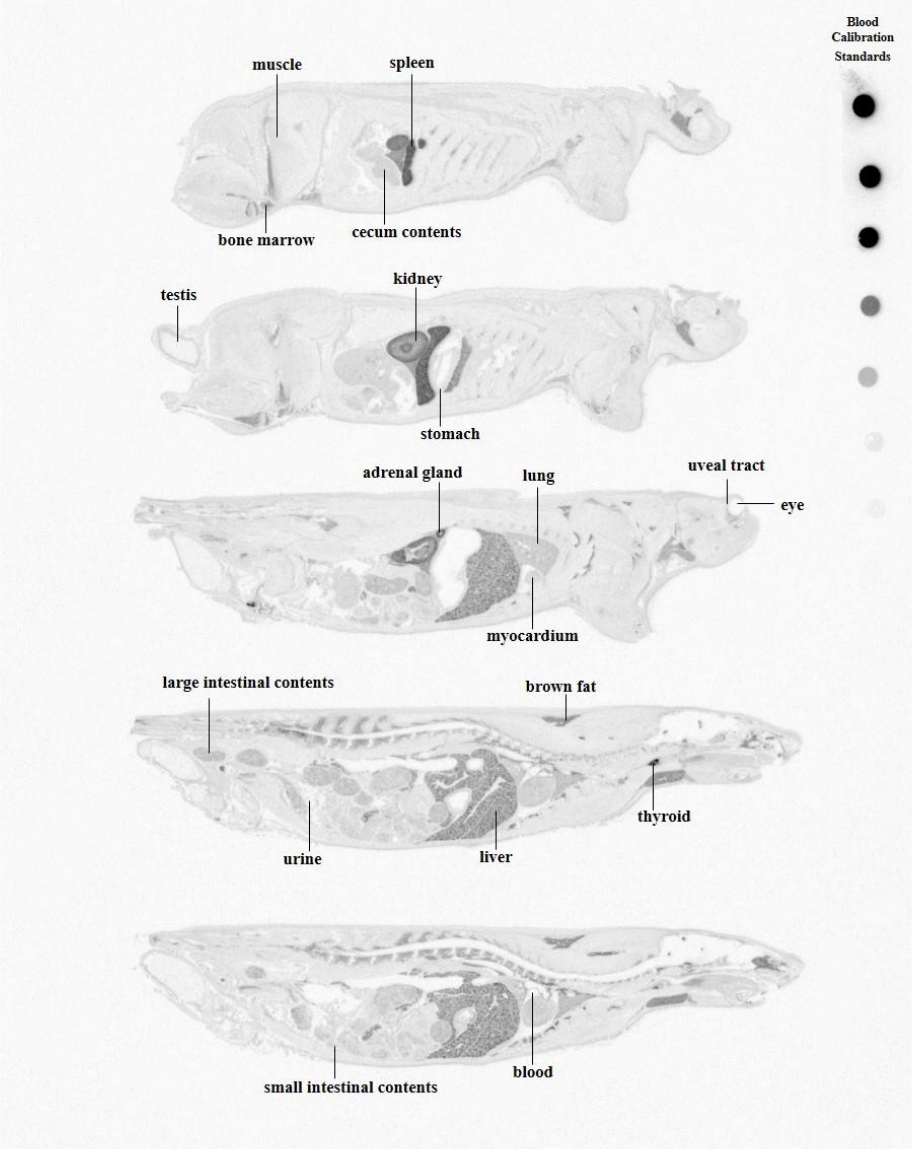

Note: Cecum and large intestinal contents are identified separately in this image; however, concentrations were averaged for quantitation.

Supplementary Figure 2. Elimination and metabolic profiles of ^{14}C-A947 following intravenous administration of ^{14}C-A947 (4 mg/kg and 200 µCi/kg) in rats. Mean cumulative percent of radioactive dose in urine and feces of intact animals (Data variability was low). Individual numerical data is listed in Supplementary Table 1c.

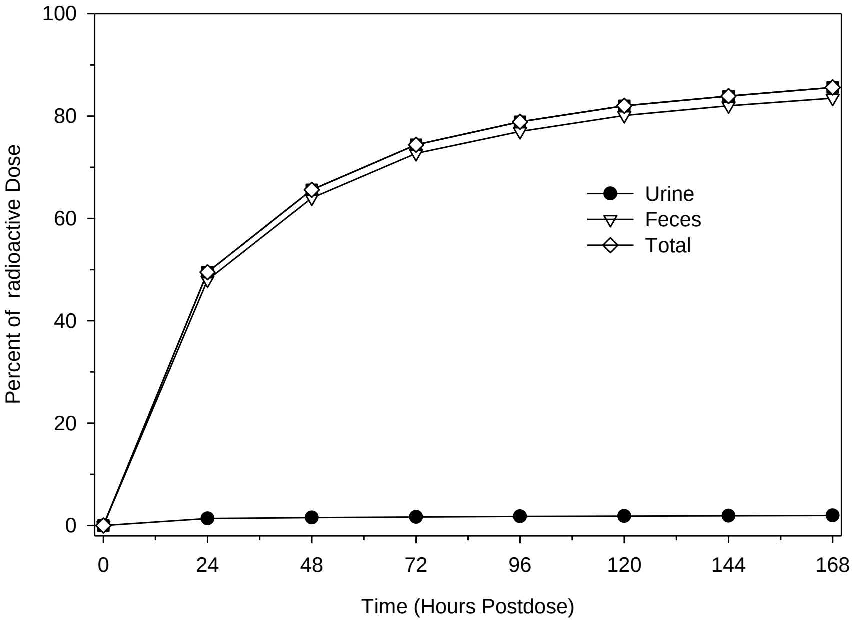

Supplementary Figure 3. Metabolic pathways of A947 in hepatocytes and liver microsomes of rats and humans.

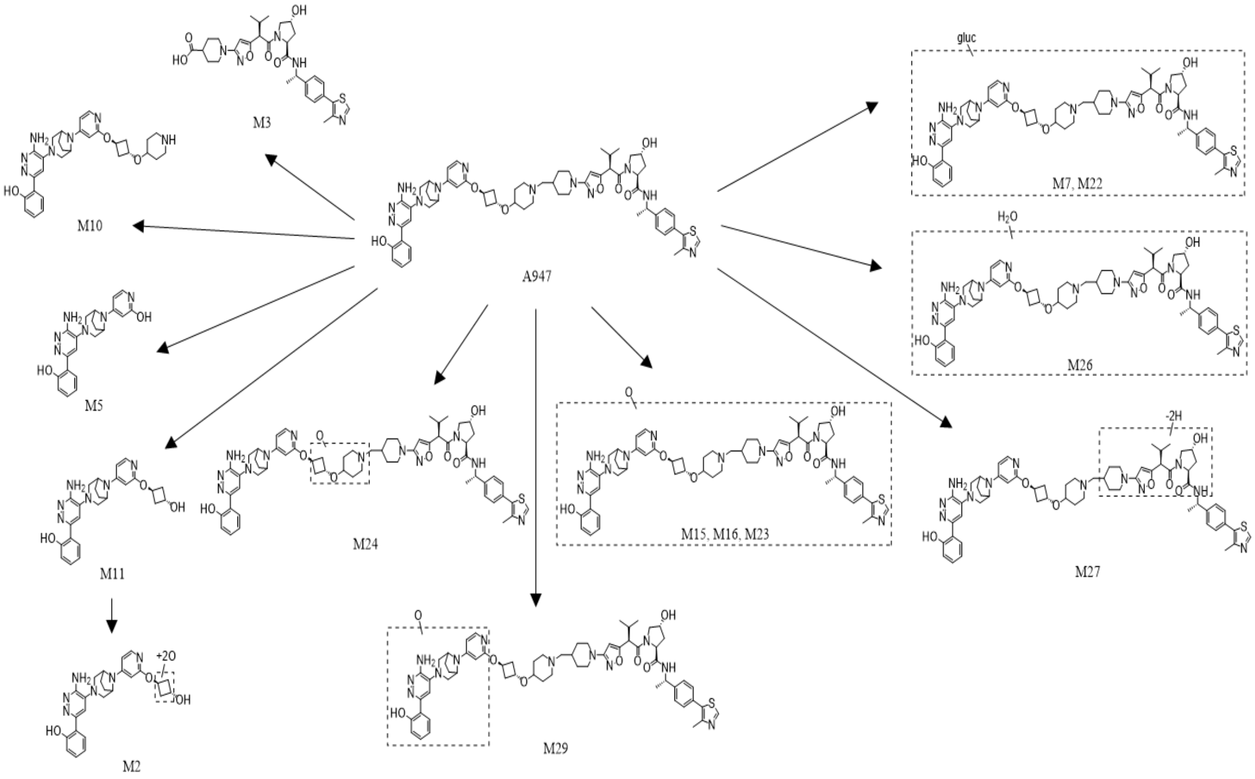

Supplementary Figure 4. Mass and NMR spectra of ^{14}C-A947.

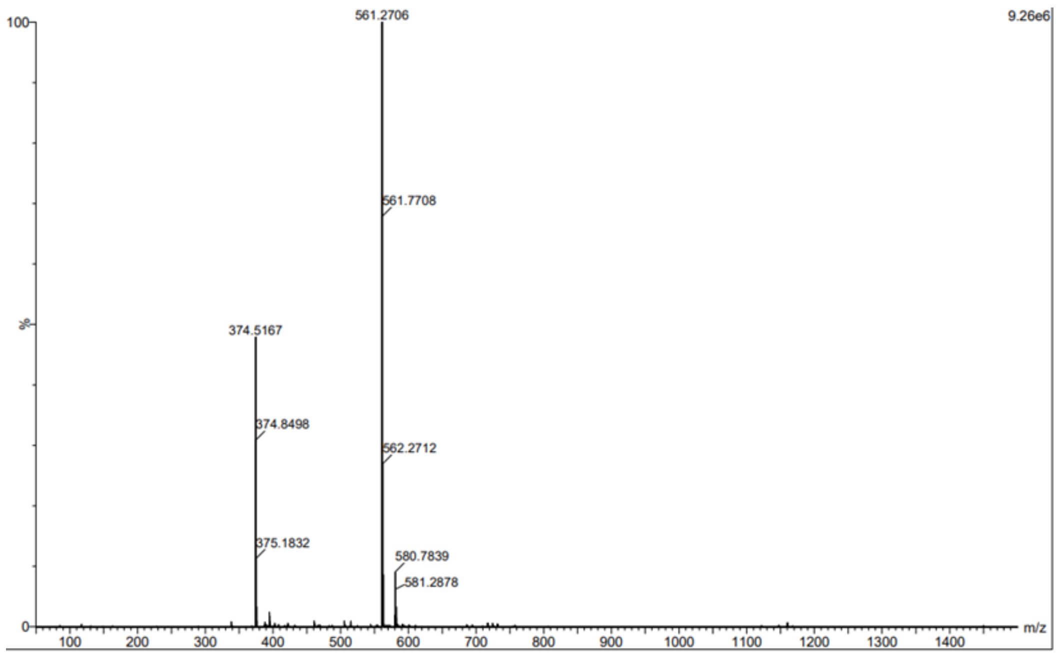

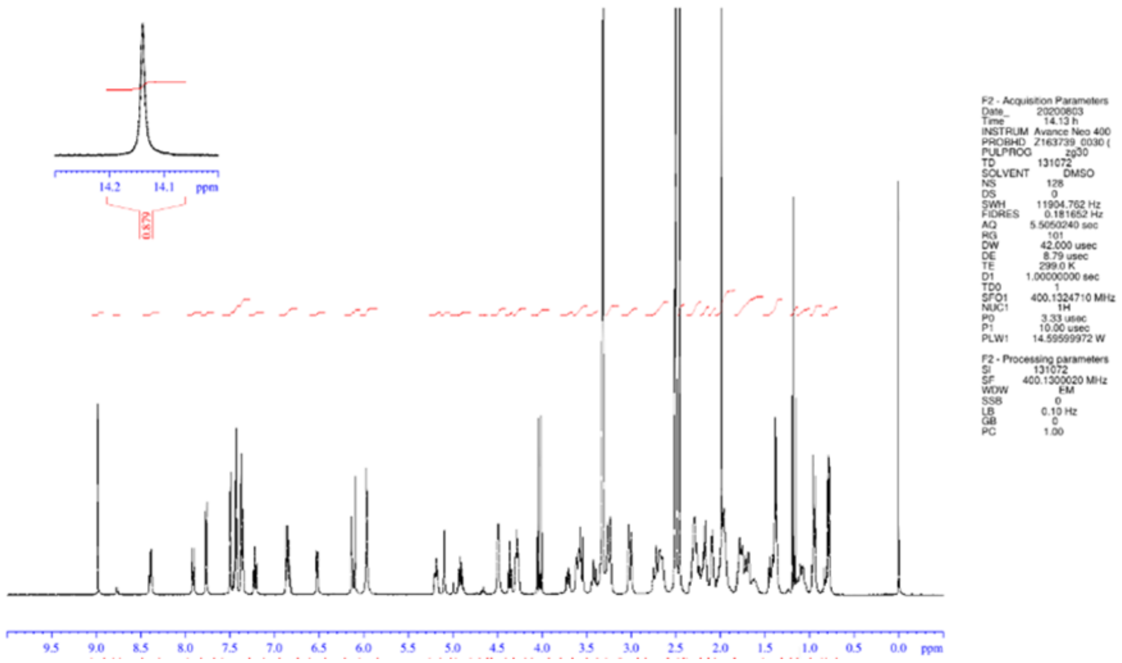

Supplement: Supplementary file 2 — Supplementary Information [file 43856_2024_505_MOESM2_ESM.pdf]
