# Supplementary material for: Tissue distribution and retention drives efficacy of rapidly clearing VHL-based PROTACs
Source: Commun Med (Lond). 2024 May 16;4:87. doi: 10.1038/s43856-024-00505-y (PMC11099041; doi:10.1038/s43856-024-00505-y)
Supplement: Supplementary file 3 — Description of Additional Supplementary Files [file 43856_2024_505_MOESM3_ESM.pdf]

# Description of Additional Supplementary Files

**File name:** Supplementary Data 1.

**File Description:** Concentrations of radioactivity in blood and tissues determined by quantitative whole body autoradiography at specified times after intravenous administration of 14C-A947 to rats (4 mg/kg, 200 µCi/kg)

**File name:** Supplementary Data 2.

**File Description:** Tissue: plasma concentration ratios by quantitative whole body autoradiography at specified times after intravenous administration of $^{14}$C- A947 to rats (4 mg/kg, 200 µCi/kg)

**File name:** Supplementary Data 3.

**File Description:** Pharmacokinetic parameters for radioactivity in blood and tissues determined by quantitative whole body autoradiography from rats after intravenous administration of $^{14}$C-A947 (4 mg/kg, 200 µCi/kg)

**File name:** Supplementary Data 4.

**File Description:** A947 Concentrations (µM) in Blood, Kidney, Liver and Lung following 1 mg/kg and 20 mg/kg intravenous Administration of A947 to Mice – on a separate file.
